# Derivatization of pBACpAK entrapment vectors for enhanced mobile genetic element transposition detection in multidrug-resistant Escherichia coli

**DOI:** 10.1099/acmi.0.001013.v3

**Published:** 2025-05-23

**Authors:** Supathep Tansirichaiya, Wasawat Leartsiwawinyu, Nattharee Thanawan, Richard N. Goodman, Chanwit Tribuddharat, Adam P. Roberts

**Affiliations:** 1 Department of Microbiology, Faculty of Medicine Siriraj Hospital, Mahidol University, Bangkok, Thailand; 2 Army Institute of Pathology, Royal Thai Army Medical Department, Bangkok, Thailand; 3 Department of Tropical Disease Biology, Liverpool School of Tropical Medicine, Pembroke Place, Liverpool, L3 5QA, UK

**Keywords:** antimicrobial resistance, *Escherichia coli*, insertion sequences, mobile genetic elements, pBACpAK entrapment vector, transposons

## Abstract

**Aim.** Antimicrobial resistance poses a critical global health threat, driven by the dissemination of resistance genes via mobile genetic elements (MGEs). This study aims to enhance the detection of MGE insertions in multidrug-resistant *Escherichia coli* by derivatizing the pBACpAK entrapment vector.

**Methods and results.** Three derivatives were constructed with additional nucleotides upstream of the *cI* repressor gene, based on conserved regions identified from GenBank sequences containing known IS*26* and IS*1* insertions. Using colony PCR, intracellular transposition screening was performed on 194 tetracycline-resistant colonies from four *E. coli* ESI123 strains carrying different pBACpAK constructs. The derivatives showed increased MGE capture rates (10.7–73.1 %) compared to the WT vector (3.75%), identifying multiple MGEs, including the novel composite transposon Tn*7824*. Tn*7824* harbours the *bla*_OXA-181_ carbapenem resistance gene and the *qnrS1* quinolone resistance gene, highlighting the clinical relevance of these findings. Long-read sequencing of transposants confirmed the accuracy of MGE identification and structural characterization, which also revealed chromosomal integration events of the pBACpAK derivatives mediated by flanking insertion sequences.

**Conclusions.** The modifications introduced in the pBACpAK derivatives could increase the detection of transposition events by alleviating spatial constraints, allowing for more robust MGE detection.

**Impact Statement**

These findings highlight the utility of entrapment vectors for studying mobile genetic element (MGE)-associated antimicrobial resistance (AMR) dissemination in clinical and environmental settings. By improving the detection of novel and clinically relevant MGEs, such as Tn*7824*, this approach contributes to a better understanding of resistance gene mobility and may aid future AMR surveillance efforts.

## Data Availability

All whole-genome sequencing data generated in this study have been deposited in the National Center for Biotechnology Information GenBank under BioProject ID PRJNA1224291. The accession numbers for each isolate (SAMN46856268 to SAMN46856290) in this BioProject are provided in Table S2. The sequences of Tn*7824* and the pBACpAK derivatives have been deposited under accession numbers PV132339 and PV591341-PV591343, respectively.

## Introduction

Antimicrobial resistance (AMR) poses a significant global public health challenge, with an estimated 1.27 million deaths attributed to resistant bacterial infections in 2019, surpassing mortality rates from human immunodeficiency virus (HIV)/AIDS and malaria [1]. Recent projections estimated that by 2050, antimicrobial resistance could lead to ~1.91 million deaths attributable to AMR and 8.22 million deaths associated with AMR annually [2,3]. The pervasive use of antimicrobials across sectors such as healthcare, agriculture and veterinary medicine exerts selective pressure, driving the development and dissemination of AMR among bacterial populations [4,5]. Nosocomial infections by the ESKAPEE pathogen group are notorious for causing severe, prolonged outbreaks in clinical settings due to their resistance to multiple antibiotic classes. For example, *Escherichia coli* alone accounted for ~800,000 AMR-related deaths in 2019 [1]. The World Health Organization has categorized carbapenem-resistant and third-generation cephalosporin-resistant *Enterobacteriaceae*, including *E. coli*, as critical priority pathogens due to the urgent need for the development of new antibiotics to combat these highly resistant bacteria [6].

A crucial factor in the accumulation and spread of antibiotic resistance genes (ARGs) in pathogens is the activity of mobile genetic elements (MGEs), which are segments of DNA capable of movement within and between genomes. Conjugative plasmids and transposons facilitate the intercellular transfer of ARGs, while insertion sequences (ISs) and transposons enable intracellular transposition between and within replicons within the same cell. Notable MGEs carrying ARGs against last-resort antibiotics have been identified in *E. coli*, such as the Tn*3000* composite transposon carrying the *bla*_NDM-1_ carbapenem resistance gene [7] and the IS*Apl1* composite transposon with the *mcr-1* colistin resistance gene [8].

To manage AMR efficiently, the identification and characterization of ARG-carrying MGEs are crucial. Traditionally, MGEs are identified through phenotypic changes conferred by accessory genes like ARGs or by comparative analysis of whole-genome sequencing (WGS) data against existing databases. However, these methods fall short in providing information on MGE transposition and face challenges in contextualizing ARGs within functional MGEs from short-read sequencing data, particularly when multiple similar MGE copies are present in bacterial genomes.

Another method involves using entrapment vectors to capture MGEs based on their transposition activity. These vectors include a genetic system that confers a selective phenotype when MGEs transpose into a specific DNA region [9]. Previously, a single copy number entrapment vector has been developed called pBACpAK, demonstrating its ability to detect MGE insertions in both laboratory and clinical *E. coli* isolates [10,12]. The pBACpAK vector contains a *cI-tetA* gene system, where the λ repressor (encoded by *cI*) constitutively inhibits the expression of the *tetA* gene by binding to the P_RM_ promoter, blocking *tetA* expression. When an MGE inserts into the *cI* gene, it disrupts the repressor’s expression, leading to the expression of *tetA* and resulting in a selectable tetracycline resistance phenotype, which allows for the detection of rare transposition events within large populations of bacteria. Derivatives of pBACpAK were also developed by replacing the selective marker from *catA1* resistance gene with *mcr-1* in pBACpAK-COL and *aph(3′)-Ia* in pBACpAK-KAN, allowing the detection of transposition in a wider range of clinical isolates by circumventing their existing resistance profiles [13]. This approach allowed the capture of multiple IS elements, novel transposons and a novel translocatable unit.

In this study, we aim to enhance the detection of MGEs in multidrug-resistant *E. coli* clinical isolates by derivatizing the original pBACpAK vector. This was achieved by adding additional nucleotides between the *cI* repressor and *tetA* genes, based on the common target sequences observed at IS*1* and IS*26* insertion sites in sequences deposited in GenBank. Both IS elements are among the most prevalent IS elements associated with ARGs in clinical multidrug-resistant *E. coli* [14,16]. This approach is designed to improve our understanding of MGE transposition dynamics and contribute to the broader effort of combating AMR. By increasing the detection sensitivity for MGE transposition, we hope to gain deeper insights into their role in the dissemination of resistance genes among pathogenic bacteria and to be able to utilize them as tools.

## Methods

### Bacterial strains, plasmids and culture conditions

All bacterial strains used in this study are listed in Table 1. These strains were cultured at 37 °C in Luria–Bertani (LB) medium with appropriate antibiotics (Sigma-Aldrich, Thailand), with the concentrations of antibiotics as follows: chloramphenicol at 12.5 µg ml^−1^, ampicillin at 100 µg ml^−1^ and tetracycline at 10 µg ml^−1^. The extended-spectrum *β*-lactamase (ESBL) *E. coli* ESI123 clinical isolate was obtained from the routine bacteriology laboratory at Siriraj Hospital, Bangkok, Thailand. The sequence type of *E. coli* ESI123 was identified using the Achtman scheme via PubMLST [17], and the putative pathovar was inferred based on the presence of virulence-associated genes using VirulenceFinder 2.0 [18]. Siriraj Hospital Institutional Review Board (IRB) reviewed this study and confirmed that ethical approval was not required as it is classified as non-human subject research. The protocols in this study were approved for biosafety by the Siriraj Safety and Risk Management Taskforce of Mahidol University (SI2023-006).

**Table 1.** Bacterial strains used in this study

Strain	Characteristic	Resistance phenotype*	Reference
**Bacterial strains**			
*E. coli* DH5α	- Chemically competent cells	–	New England Biolabs
*E. coli*::pBACpAK-WT	- *E. coli* MDS strain containing the original pBACpAK entrapment vector from the previous study	Chl^R^	[10]
*E. coli* ESI123	- Clinical strain isolated in 2021	Amp^R^	This study
*E. coli* ESI123::pBACpAK-WT	*- E. coli* ESI123 containing pBACpAK-WT	Chl^R^, Amp^R^	This study
*E. coli* ESI123::pBACpAK-16bp-3Prime	*- E. coli* ESI123 containing pBACpAK-16bp-3Prime	Chl^R^, Amp^R^	This study
*E. coli* ESI123::pBACpAK-16bp-5Prime	*- E. coli* ESI123 containing pBACpAK-16bp-5Prime	Chl^R^, Amp^R^	This study
*E. coli* ESI123::pBACpAK-26bp	*- E. coli* ESI123 containing pBACpAK-26bp	Chl^R^, Amp^R^	This study
**Plasmids**			
pBACpAK-WT	- Entrapment vector from previous study	Chl^R^	[11]
pBACpAK-16bp-3Prime	- pBACpAK vector with the addition of 16 bp derived from the 3′ end of the curated IS*26* insertion sites	Chl^R^	This study
pBACpAK-16bp-5Prime	- pBACpAK vector with the addition of 16 bp derived from the 5′ end of the curated IS*26* insertion sites	Chl^R^	This study
pBACpAK-26bp	- pBACpAK vector with the addition of 26 bp derived from the 3′ end of the curated IS*1* insertion sites	Chl^R^	This study

*ChlR, chloramphenicol resistance; AmpR, ampicillin resistance.

### Construction of the MGE-targeted pBACpAK entrapment vectors

**Fig. 1.**
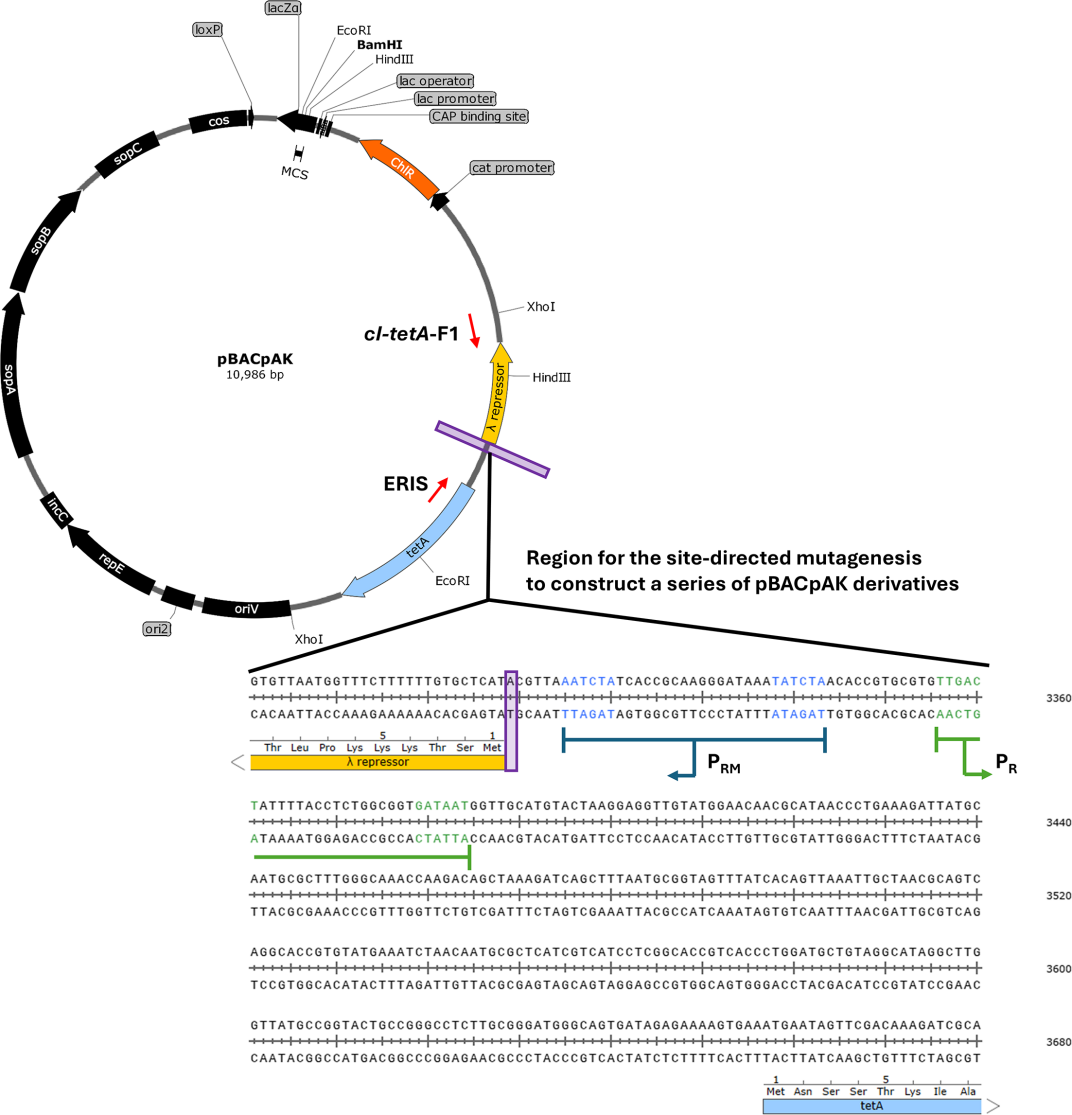
pBACpAK and location for constructing the pBACpAK derivatives. The *cI* repressor, chloramphenicol, tetracycline and other genes are represented by yellow, orange, blue and black open-arrowed boxes, respectively. The additional nucleotides were added at the start of the *cI* repressor gene, indicated by the purple boxes. The red arrows indicate the positions and orientations of the primers used for colony PCR: *cI-tetA*-F1 (forward) and ERIS (reverse), which amplify the region from the end of the *c*I repressor gene to the start of *tetA*.

A series of entrapment vectors was constructed based on the pBACpAK vector to capture MGEs of interest. This was done by adding the putative target sites of MGEs associated with ARGs from the selected clinical isolates between the *cI* and *tetA* genes of pBACpAK (Fig. 1). The insertion sites for each MGE were identified by curating sequences flanking the MGEs of interest from the GenBank DNA database. These sequences were then aligned using clustal omega [19] to determine the putative consensus insertion sites for each MGE through WebLogo [20].

The MGE insertion site sequences were incorporated between the *cI* and *tetA* genes of pBACpAK via site-directed mutagenesis (Fig. 1). Target sequences and a NheI restriction site (GCTAGC) were added to the forward and reverse primers designed using NEBaseChanger. PCR was performed using pBACpAK as a DNA template. The resulting PCR amplicons were treated with a kinase–ligase–DpnI (KLD) enzyme mix and then transformed into *E. coli* DH5α competent cells. Clones with the MGE-targeted-pBACpAK were confirmed by NheI digestion and Sanger sequencing across the target site between the *cI* and *tetA* genes.

### Preparation and electroporation of *E. coli* electrocompetent cells

*E. coli* clinical isolates were prepared as electrocompetent cells following a previously described protocol [21]. An overnight culture was grown in LB broth supplemented with ampicillin (selective markers for ESBL*–E. coli* clinical isolates) and chloramphenicol (a selective marker for pBACpAK). This overnight culture was then diluted to an OD_600_ of 0.05 in a flask containing 50 ml of LB broth and incubated until it reached the mid-exponential phase (OD_600_ of 0.6). The culture was split into two 50-ml tubes and incubated on ice for 10 min. Cells were pelleted by centrifugation at 4 °C and 2,500 ***g*** for 10 min, after which the supernatant was discarded. The pellet was resuspended in 20 ml of pre-chilled 10% glycerol in distilled water and washed three times. Finally, the pellet was resuspended in 10% glycerol, aliquoted into 50 µl portions in pre-chilled cryotubes and stored at −80 °C.

In a pre-chilled 1.5-ml microcentrifuge tube, 50 µl of electrocompetent cells were mixed with 10–100 ng of pBACpAK plasmids, and the mixture was transferred to a pre-chilled 0.1-cm electroporation cuvette. Electroporation was performed with the settings of 1.8 kV, 200 Ω and 25 µF. After electroporation, 950 µl of pre-warmed SOC medium was added to the cells, and the mixture was transferred to a fresh 50-ml tube and incubated at 37 °C for 1 h. The transformants were then plated on LB agar supplemented with chloramphenicol (to select for pBACpAK) and ampicillin (to select for clinical isolates) to select for successful transformants.

### Screening for the transformants with MGE insertions on pBACpAK

The *E. coli* transformants carrying pBACpAK derivatives were subcultured into 5 ml of LB medium supplemented with chloramphenicol and ampicillin and incubated at 37 °C for 4 h. A 500 µl aliquot of this culture was then plated on LB agar supplemented with the same antibiotics and tetracycline (MGE screening agar). The remaining culture continued to incubate overnight, after which a 100 µl aliquot was plated on MGE screening agar. This process was repeated daily, with the overnight culture used to inoculate new 5 ml LB broth, followed by plating and subculturing for an additional 3 days. Plates were monitored for colony growth every day for a week, and all resulting colonies were subcultured onto fresh MGE screening agar to confirm their tetracycline resistance phenotype.

All confirmed tetracycline-resistant clones were initially screened for MGE insertions to confirm they are transposants [12] in the *cI-tetA* region of pBACpAK using colony PCR with *cI-tetA*-F1 and ERIS primers. The colony PCR was conducted with standard DNA polymerase, which can amplify up to 6 kb, to eliminate clones with mutations, insertions, deletions and duplications. Colony PCR was performed using a 10 µl reaction consisting of 5 µL of 2×MyTaq Red Master Mix (Bioline, UK), 1 µl of each primer (10 µM) and 3 µl of molecular-grade water. PCR conditions were as follows: initial denaturation at 95 °C for 10 min; followed by 35 cycles of 95 °C for 1 min, 56.5 °C for 30 s and 72 °C for 3 min; and with a final extension at 72 °C for 5 min.

Any *cI-tetA* amplicons larger than the WT *cI-tetA* amplicon (1.35 kb) were sequenced using the Sanger sequencing service from BNK Bioscience, Thailand. Clones that failed to amplify a product with *cI-tetA*-F1 and ERIS primers were analysed by sequencing through both short- and long-read sequencing. Short-read sequencing was performed by Getz Healthcare, Thailand, using the Illumina HiSeq/NovaSeq platform with a PE150 protocol (2×150 bp), while long-read sequencing was performed by using Oxford Nanopore Technologies at Siriraj Translational Microbial Genomics and Data Center (SiTMiD) using MinION with the R10.4.1 flow cell.

### Bioinformatics analysis on transposant genomes

The short-read sequencing data was processed using AfterQC to trim and filter low-quality reads (e.g. *Q*<20). For the long-read sequencing data, the Guppy algorithm (version 5.0.7) [22] was used for the base calling with the HAC (high accuracy) model to convert the raw signals FAST5 to Fastq files; then the adapter was trimmed by Porechop version 0.2.4 [23]. Assembly was then performed by using the Unicycler version 0.4.8 for hybrid assembly and Flye version v2.9.5 [24,25] for long-read-only assembly. The data from the Sanger sequencing and hybrid assembly were analysed using blastn, blastx and ISFinder to determine similar matches in the nucleotide, protein and IS element databases, respectively [26,27].

### Deposition of novel transposons and WGS data of transposants

A novel transposon was assigned Tn numbers by The Transposon Registry [28] and designated as Tn*7824* (accession number PV132339). The WGS data have been deposited in the National Center for Biotechnology Information (NCBI) database with accession numbers ranging from SAMN46856268 to SAMN46856290. In addition, the annotated sequences of the modified pBACpAK entrapment vectors have been deposited in GenBank under accession numbers PV591341–PV591343.

## Results

### Construction of the pBACpAK derivatives and introduction into *E. coli* clinical strains

Three derivatives of the pBACpAK entrapment vector were constructed by adding sequences of 16 and 26 bp upstream of the *cI* gene. These additional nucleotides were incorporated through primers (listed in Table S1, available in the online Supplementary Material) and constructed using the Q5 site-directed mutagenesis kit. The 16 bp sequences consisted of the 10 bp most commonly found at the 5′ and 3′ ends of the IS*26* insertion site, along with the NheI restriction site (Fig. S1). These were named pBACpAK-16bp-5prime and pBACpAK-16bp-3prime, respectively. The 26 bp derivative, called pBACpAK-26bp, included the 20 bp sequence most commonly found at the 5′ end of the IS*1* insertion site along with the NheI restriction site. The constructed derivatives were cloned into *E. coli* DH5α and confirmed by Sanger sequencing across the *cI-tetA* region.

The ESBL *E. coli* strain ESI123 was selected as a model to test the pBACpAK derivatives, as it was sensitive to tetracycline and chloramphenicol, the antibiotics used for screening. WGS analysis of *E. coli* ESI123 revealed that it belongs to sequence type ST410 based on the Achtman MLST scheme, a globally distributed extraintestinal pathogenic *E. coli* lineage commonly associated with antimicrobial resistance [29]. Screening with VirulenceFinder identified the presence of *fimH*, *fyuA* and *fdeC*, consistent with a uropathogenic *E. coli* pathotype [30]. The isolate was obtained from a urine sample via an indwelling catheter, indicating a probable urinary tract infection.

All pBACpAK derivatives, including pBACpAK-WT (the original pBACpAK), pBACpAK-16bp-5prime, pBACpAK-16bp-3prime and pBACpAK-26bp, were introduced through electroporation, as listed in Table 1. The inserted sequence in pBACpAK-16bp-5prime site was 5′-GCTAGCGTACTCTAAA-3′, pBACpAK-16bp-3prime site was 5′-GCTAGCTTTAGAGTAC-3′ and pBACpAK-26bp site was 5′-GCTAGCAATATAACCACGATAAGGTA-3′, introduced upstream of the *cI* repressor gene.

### Determining the efficiency of the newly constructed pBACpAK derivatives

*E. coli* ESI123 containing pBACpAK derivatives was subcultured and screened for colonies with a tetracycline resistance phenotype. The number of tetracycline-resistant colonies screened from each strain and the number of confirmed transposants are shown in Table 2. It was shown that 3 out of 80 (3.75 %) tetracycline-resistant *E. coli* ESI123::pBACpAK-WT colonies were identified with MGE insertion into the pBACpAK-WT vector. In contrast, the newly constructed pBACpAK derivatives exhibited significantly higher percentages of transposants: 10.7% for pBACpAK-16bp-5Prime, 37.5% for pBACpAK-16bp-3Prime and 73.1% for pBACpAK-26bp. These represent approximately 2.9-fold, 10-fold and 19.5-fold increases in transposition detection efficiency compared to the WT pBACpAK.

**Table 2.** The number of tetracycline-resistant colonies and transposants found from each pBACpAK derivative

Strains::entrapment vector	Tetracycline-resistance colony*	No. of transposant			
		>2 kb insertion	<2 kb insertion	Total (colony)	% Transposant
*E. coli* ESI123::pBACpAK-WT	80	1	2	3	3.75%
*E. coli* ESI123::pBACpAK-16bp-3Prime	32	6	6	12	37.5%
*E. coli* ESI123::pBACpAK-16bp-5Prime	56	6	0	6	10.7%
*E. coli* ESI123::pBACpAK-26bp	26	2	22	19	73.1%

*All tetracycline-resistant colonies were screened by colony PCR across the *cI-tetA* region. Wt-sized amplicons indicated the absence of detectable MGE insertion or integration.

### Characterization of the MGE insertions in transposants

**Fig. 2.**
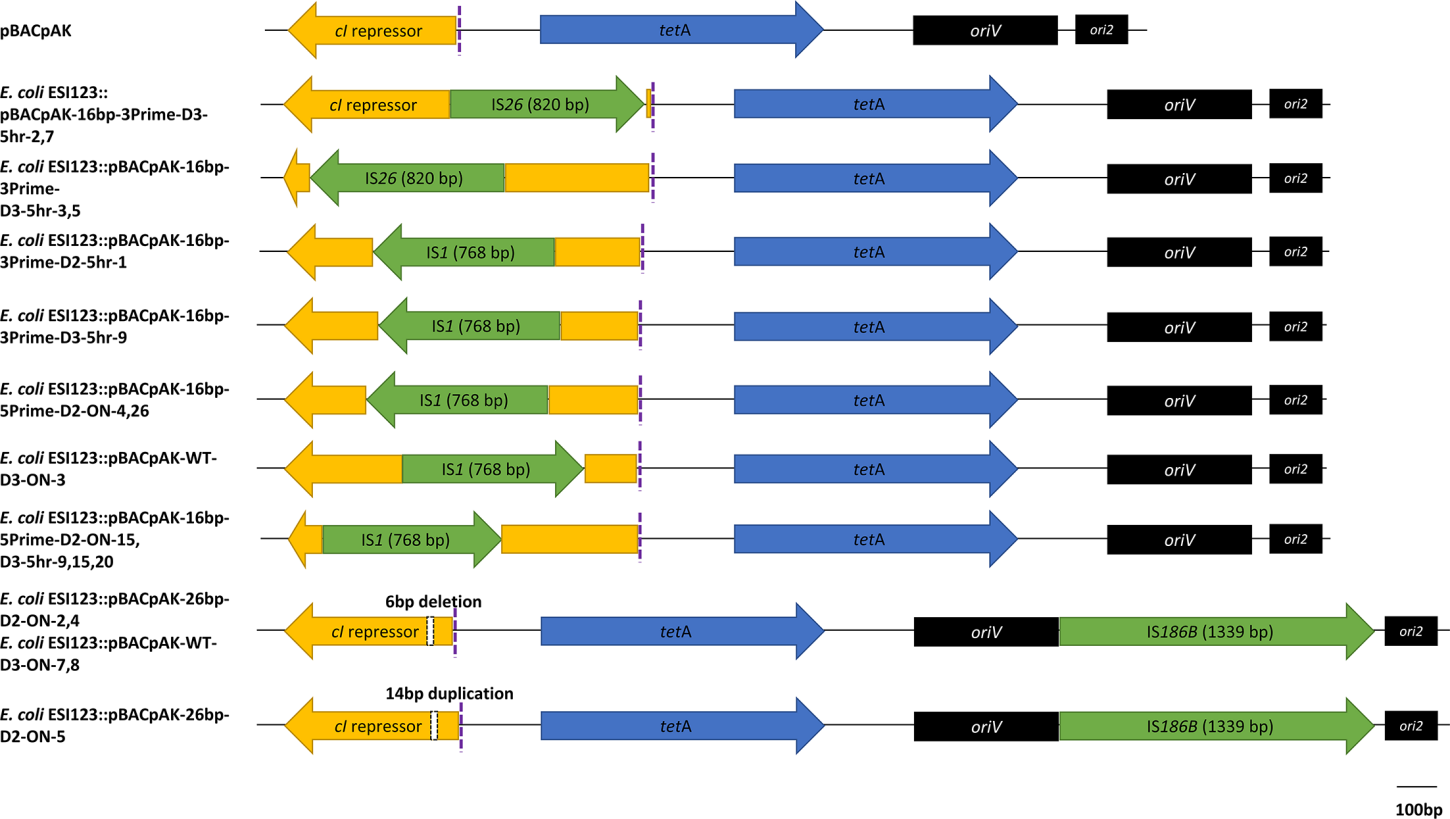
Transposants with less than 2 kb insertion. The *cI* gene, MGEs, *tetA* and other genes are represented by yellow, blue, green and black open-arrowed boxes, respectively. The additional nucleotides were added at the start of the *cI* repressor gene, indicated by the dashed purple lines.

Among all transposants, 13 showed an insertion of less than 2 kb into the *cI-tetA* region, which were subsequently characterized by Sanger sequencing. The analysis revealed the insertion of two IS elements: IS*1*, in both pBACpAK-WT and the pBACpAK derivatives, and IS*26* in the pBACpAK-16bp-3Prime entrapment vector. Another five colonies showed a 6 bp deletion and 14 bp duplication within the *cI-tetA* region and had an insertion of an IS*186B* between the *oriV* and the *ori2* of the pBACpAK-WT and the pBACpAK-26bp vector. Surprisingly, none of the insertion sites of any sequenced clones were located in the extra nucleotides added to the pBACpAK-WT. Based on the IS elements, insertion direction and sites, they were categorized into eight different transposition events, as shown in Fig. 2.

**Fig. 3.**
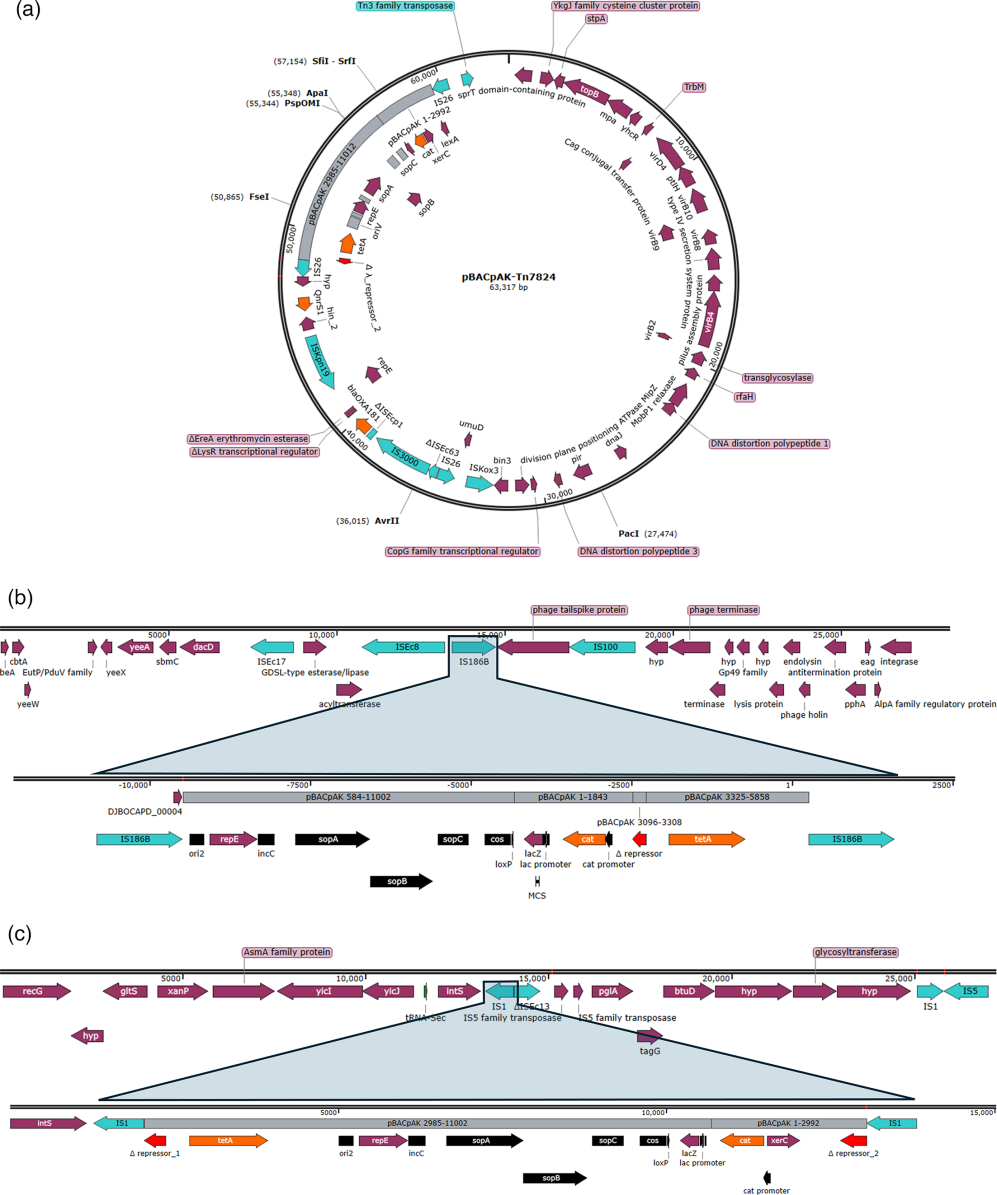
Transposant cointegrates with the 52 kb plasmid and chromosomal DNA of *E. coli* ESI123 clinical isolate. (**a**) The cointegrate between the pBACpAK derivative and the 52 kb plasmid resulted in the formation of Tn*7824*, flanked by IS*26*. (**b**) and (c) The integration of pBACpAK derivative into the host chromosomal DNA flanked by IS*1* and IS*186B*, respectively.

The transposants that exhibited no amplicon in the colony PCR screening, often indicating larger insertions, were subsequently characterized through WGS. Among these, 15 out of 28 showed an insertion of an entire 52 kb plasmid in the *cI-tetA* region, flanked by two copies of IS*26* on each side. This 52 kb plasmid carried by the *E. coli* ESI123 clinical isolate had 99% similarity to the plasmid pJBCDAAC-19–0068_OXA-181 previously isolated from *E. coli* (accession number LC778280) [31]. The structure of the captured element found in these clones conformed to the definition of a composite transposon; thus, it was annotated as Tn*7824*. It contains the carbapenemase resistance gene *bla*_OXA-181_, the quinolone resistance gene *qnrS1* and multiple insertion sequences including IS*26*, IS*3000*, IS*Kpn19* and IS*Kox3*. Based on the insertion site and orientation of IS*26*, these Tn*7824*-containing pBACpAK transposants were classified into five different transposition groups, as shown in Table 3. Fig. 3 shows the structure of the Tn*7824*-pBACpAK cointegrate.

**Table 3.** The details of MGEs captured by pBACpAK derivatives

Bacterial strain(accession no.)	MGE/mutation	Location on pBACpAK (position)	Size (bp)	Percentage of similarity (ISFinder/NCBI)		Direct repeat*
				blastn	Accession no.	
***E. coli* ESI123::pBACpAK-WT**						
D0-ON-3	IS*1*	*cI* repressor (3089)	768	99.7%	X52534	GCTAACTTT
D0-ON-7,8	Deletion	*cI* repressor (3177)	6	–	–	–
	IS*186B*	Between *oriV* and *ori2* (5831)	1,338	99.9%	X03123	GGGGTGCCCCC
***E. coli* ESI123::pBACpAK-16bp-5Prime**						
D2-ON-4,26	IS*1*	*cI* repressor (2933)	768	99.7%	X52534	CACCTTTGG
D2-O/N-15, D3-5hr-9,15,20	IS*1*	*cI* repressor (2731)	768	99.7%	X52534	TTGAAGGTA
***E. coli* ESI123::pBACpAK-16bp-3Prime**						
D3-5hr-2,7	IS*26*	*cI* repressor (3294)	820	99.9%	X00011	CTTTTTTG
D3-5hr-3,5	IS*26*	*cI* repressor (2700)	820	99.9%	X00011	AAAACACC
D2-5hr-1	IS*1*	*cI* repressor (2951)	768	99.6%	X52534	TAAGCTCAG
D3-5hr-9	IS*1*	*cI* repressor (2985)	768	96.4%	X52534	AGAAAAAA
4hrTet-CAT5 −9,10,12	Deletion	*cI* repressor (1844)	1,253	–	–	–
	Deletion	*cI* repressor (3308)	17	–	–	–
	Chromosomal DNA integration flanked by two IS*186B* elements	Between *oriV* and *ori2* (5831)	1,338	99.9%	X03123	GGGGTGCCCCC
4hrTet-CAT5-8	Tn*7824*(similar to pJBCDAAC-19–0068_OXA-181)	*cI* repressor (2699)	52,299	100%	LC778280	AAAACACC
D2-5hr-4	Tn*7824*(similar to pJBCDAAC-19–0068_OXA-181)	*cI* repressor (2986)	52,299	100%	LC778280	GTTTTTTC
D1-ON-18, Tet10-4hr-5	Chromosomal DNA integration flanked by two IS*1* elements	*cI* repressor (2985)	768	99.7%	X52534	TTTTTTCT
***E. coli* ESI123::pBACpAK-26bp**						
D2-ON-2,4	Deletion	*cI* repressor (2996)	6	–	–	–
	IS*186B*	Between *oriV* and *ori2* (5831)	1,338	99.9%	X03123	GGGGTGCCCCC
D2-ON-5	Duplication	*cI* repressor (2963)	14	–	–	–
	IS*186B*	Between *oriV* and *ori2* (5831)	1,338	99.9%	X03123	GGGGTGCCCCC
D3-5hr-1,6	Tn*7824* (similar to pJBCDAAC-19–0068_OXA-181)	*cI* repressor (2806)	52,299	100%	LC778280	TTAATTCT
D2-5hr-2, D3-ON-1,2,3,4, D3-5hr-3,	Tn*7824*(similar to pJBCDAAC-19–0068_OXA-181)	*cI* repressor (2985)	52,299	100%	LC778280	TTTTTTCT
D1-ON-1, D1-5hr-1,4	Tn*7824* (similar to pJBCDAAC-19–0068_OXA-181)	*cI* repressor (3000)	52,299	100%	LC778280	AGTATGAG

*The direct repeat represents the target site duplication formed upon insertion of the MGEs identified in each isolate.

Additionally, five colonies were found where their pBACpAK derivatives were integrated into *E. coli* chromosomal DNA, flanked by insertion sequences. In *E. coli* ESI123::pBACpAK-16bp-3Prime-D1_ON_18 and D0_4 h_5, pBACpAK recombined with chromosomal DNA within the *cI* gene, resulting in the separation of the *cI* gene on each site, flanked by two IS*1* elements. In *E. coli* ESI123::pBACpAK-16bp-3Prime-D0-4hr-9, 10 and 12, the pBACpAK was flanked by two copies of IS*186B*, with recombination occurring between *oriV* and *ori2*, accompanied by a 1.4 kb deletion in the *cI* gene.

## Discussion

The results of our study underscore the critical role of MGEs in the dissemination of AMR genes among pathogenic *E. coli* strains. Our findings align with previous reports that MGEs, such as ISs and transposons, facilitate the transposition of resistance determinants, thereby exacerbating the AMR crisis. The construction and utilization of the pBACpAK derivatives, particularly the pBACpAK-16bp-5Prime, pBACpAK-16bp-3Prime and pBACpAK-26bp vectors, demonstrated a higher efficiency in capturing MGE insertions compared to the WT pBACpAK vector. This suggests that the additional nucleotides in these derivatives enhance the detection sensitivity for MGEs. Notably, the insertion rates ranged from 10.7 to 73.1% of tetracycline-resistant colonies, indicating the robustness of these derivatives in identifying active transposition events.

All tetracycline-resistant colonies were screened by colony PCR to rule out those lacking MGE transposition or chromosomal integration. Colonies that produced WT-sized amplicons likely represent derepression of the *tetA* gene through spontaneous mutations in the *cI* repressor, minor insertions or deletions and small duplications that did not involve canonical transposition events. The relatively high number of tetracycline-resistant colonies investigated for the WT pBACpAK vector, despite its lower transposant percentage of confirmed transposants, could be attributed to the need for a larger screening pool to detect rare insertion events. This approach was taken to ensure transposition activity could be captured, even at low efficiency, and to confirm that the vector remained functionally capable of detecting MGEs.

Characterization of the MGEs within the transposants revealed a diversity of captured insertion sequences, including IS*1*, IS*26* and IS*186B*, which were all detected by pBACpAK WT previously [10,12]. While the engineered sequences were based on IS*1* and IS*26* target site preferences, the pBACpAK derivatives are not limited to capturing those elements. The detection of IS*186B* demonstrates that these vectors can trap a broader range of MGEs beyond their design templates. In this study, a novel composite transposon Tn*7824* was captured by the pBACpAK derivatives, harbouring *bla*_OXA-181_ and *qnrS1*, which are associated with resistance to carbapenems and quinolones, respectively. Tn*7824* represents an IS26-mediated integrated plasmid, reinforcing the significance of IS*26* in facilitating the mobilization of large, multidrug-resistant plasmids. This study also represents the first application of the pBACpAK entrapment vector in Thailand, expanding its use beyond previously studied settings. Given Thailand’s high burden of AMR and its role as a regional hub for antibiotic use in healthcare and agriculture [32,33], applying this method to local bacterial isolates provides valuable insights into the dynamics of resistance gene dissemination. The successful capture of Tn*7824* further underscores the importance of using entrapment vectors to track emerging MGEs in clinical isolates from this region.

Our study suggests that the cointegration of pBACpAK and the 52 kb plasmid and the integration of the pBACpAK into the chromosomal DNA could be sequential events. This is evidenced by the presence of a single copy insertion of each IS element at the same positions in the transposants with less than 1 kb insertions, suggesting that these IS elements first transpose in and subsequently mediate the recombination between the two IS*26*-containing replicons. WGS of the WT *E. coli* ESI123 revealed a single-copy IS element at the insertion site, while two copies were found flanking the plasmid/chromosomal integration sites in these transposants, suggesting that these events could likely occur through homologous recombination or a copy-in mechanism.

The cointegration between pBACpAK and the 52 kb plasmid also illustrates how multidrug resistance plasmids can acquire additional resistance genes through the activity of MGEs, reflecting events that could occur in clinical settings. For instance, a plasmid initially carrying only carbapenemase and quinolone resistance genes could acquire additional resistance genes for chloramphenicol and tetracycline through such recombination events. This exemplifies the dynamic nature of plasmid evolution and the role of MGEs in shaping the resistance profiles of clinical pathogens.

The integration of pBACpAK derivatives into the *E. coli* chromosomal DNA, particularly within the *cI* gene and between *oriV* and *ori2*, further exemplifies the versatility and impact of MGEs. The observed recombination events, flanked by IS elements, suggest that these sequences can mediate chromosomal integration, potentially leading to stable inheritance of resistance genes. A similar mechanism was reported in a previous study, where the *mcr-1* colistin resistance gene, carried within the Tn*7511* composite transposon flanked by two IS*Apl1* elements, was transferred from the pMCR-E2899 plasmid to the chromosome of *E. coli* DH5α [10,12]. This genetic mechanism can contribute to the persistence and spread of AMR within bacterial populations, even in the absence of selective pressure from antibiotics.

Interestingly, transposition did not occur at the extra nucleotide sequences added in the construction of the novel entrapment vectors. It was hypothesized that these sequences might act as additional ribosomal binding sites, potentially increasing *cI* gene expression and creating stress conditions that promote transposition. An increase in MGE transposition activity due to stress conditions, such as antibiotic exposure and oxidative stress, has been shown by several studies [34,35]. However, no significant differences in protein expression patterns were observed between *E. coli* ESI123 WT, *E. coli* ESI123::pBACpAK-WT and *E. coli* ESI123::pBACpAK-16bp in our preliminary SDS-PAGE, as shown in Figure S2. However, the lack of observable differences does not rule out subtle effects that could promote transposition.

Adding extra nucleotides between the promoter and the *cI* repressor gene may provide additional space for the DNA polymerase to bind while still allowing the transposase to catalyse the transposition of insertion sequences into the *cI* repressor gene. In the original configuration, the space between the *cI* repressor gene and the promoter may be too short, such that when RNA polymerase binds to the promoter, there is insufficient space for the transposase to also bind and facilitate insertion. By adding 16–26 extra bp in this region, the additional space may allow the transposase to function effectively even when the RNA polymerase is bound. Consequently, the pBACpAK derivatives with these extra nucleotides can be subject to more transposition events. This adjustment potentially alleviates spatial constraints, thus enhancing the overall efficiency of MGE detection. There are examples of previous studies showing that DNA structural features, such as spacing, flexibility and local conformation, can influence transposition activity. For example, IS*1* transposition depends on spacer length, IS*26*-mediated rearrangements are shaped by local DNA structure and Tn*5* activity is affected by DNA bending [36,38]. Additionally, DNA-binding proteins like IHF can enhance IS*1* transposition by promoting DNA bending [39]. These findings suggest that spacing and accessibility, rather than specific sequences alone, may play a central role in the ability of some MGEs to successfully transpose to an insertion site within cI in the pBACpAK derivatives described here.

Our study also highlights the limitations of traditional methods for MGE identification, which often rely on phenotypic changes or bioinformatic analysis of WGS data. The entrapment vector approach employed here provides a complementary strategy that positively selects for active transposition events, offering a more comprehensive understanding of MGE dynamics. This method proved effective in detecting both well-characterized and novel MGEs, emphasizing its utility in AMR surveillance and research. Future studies could delve deeper into the underlying mechanisms, exploring whether alternative nucleotide sequences or configurations might better facilitate targeted transposition.

As a plasmid-based detection system, this approach has certain limitations. The host range may be constrained by the compatibility of the plasmid’s origin of replication with the bacterial species of interest. Expanding the application of these derivatives to other pathogens could further demonstrate their versatility and relevance. Our previous work has shown that the current pBACpAK system is functional in *Klebsiella pneumoniae*, suggesting broader compatibility across related taxa [10]. In addition, the current reliance on chloramphenicol and tetracycline resistance markers restricts use to strains that are susceptible to both antibiotics. However, previous studies have demonstrated that the pBACpAK system can be modified by replacing the *catA1* resistance gene with alternative markers, such as *aph(3′)-Ia* or *mcr-1*, enabling its use in strains with different resistance backgrounds [13].

In conclusion, the derivatization of the pBACpAK entrapment vectors by adding DNA sequences between the promoter and the cognate gene resulted in increased transposition events. This approach provides a refined method for detecting and characterizing MGEs in multidrug-resistant *E. coli*. These findings contribute to our understanding of the mechanisms facilitating AMR dissemination. Future research should focus on expanding the application of this approach to other clinically relevant pathogens and exploring the potential of MGE-targeted interventions to mitigate the spread of AMR.

## Supplementary material

10.1099/acmi.0.001013.v3 Fig. S1.

## Abbreviations

AMR: antimicrobial resistance
ARGs: antibiotic resistance genes
ESBL: extended-spectrum *β*-lactamase
HIV: human immunodeficiency virus
ISs: insertion sequences
LB: Luria–Bertani
MGEs: mobile genetic elements
NCBI: National Center for Biotechnology Information
WGS: whole-genome sequencing
