# Supplementary material for: Derivatization of pBACpAK entrapment vectors for enhanced mobile genetic element transposition detection in multidrug-resistant Escherichia coli
Source: Access Microbiol. 2025 May 23;7(5):001013.v3. doi: 10.1099/acmi.0.001013.v3 (PMC12102499; doi:10.1099/acmi.0.001013.v3)
Supplement: Fig. S1. [file acmi-7-01013-s001.pdf]

Supplementary materials

Derivatization of pBACpAK Entrapment Vectors for Enhanced Mobile Genetic Elements Transposition Detection in Multidrug-Resistant *Escherichia coli*

Supathep Tansirichaiya[1]*, Wasawat Leartsiwawinyu[1], Nattharee Thanawan[1,2], Richard N. Goodman[3], Chanwit Tribuddharat[1], Adam P. Roberts[3]

[1] Department of Microbiology, Faculty of Medicine Siriraj Hospital, Mahidol University, Bangkok, Thailand

[2] Army Institute of Pathology, Royal Thai Army Medical Department, Thailand

[3] Department of Tropical Disease Biology. Liverpool School of Tropical Medicine, Pembroke Place, Liverpool, L3 5QA, UK

*Corresponding author; Department of Microbiology, Faculty of Medicine Siriraj Hospital, Mahidol University, Bangkok, Thailand (Email: supathep.tan@mahidol.ac.th)

Supplementary Table 1 Primers used in this study

Primer name	Sequence (5'-3')	Gene target	Reference
**Primers for the construction of pBACpAK derivatives**			
Q5SDM_16bp_5Prime_F	ACTCTAAAACGTTAAATCTATCACCG	Construction of pBACpAK-16bp-5Prime	This study
Q5SDM_16bp_5Prime_R	ACGCTAGCATGAGCACAAAAAAGAAAC	Construction of pBACpAK-16bp-5Prime	This study
Q5SDM_16bp_3Prime_F	TAGAGTACACGTTAAATCTATCACCG	Construction of pBACpAK-16bp-3Prime	This study
Q5SDM_16bp_3Prime_R	AAGCTAGCATGAGCACAAAAAAGAAAC	Construction of pBACpAK-16bp-3Prime	This study
Q5SDM_26bp_F	CCACGATAAGGTAACGTTAAATCTATCACCG	Construction of pBACpAK-26bp	This study
Q5SDM_26bp_R	TTATATTGCTAGCATGAGCACAAAAAAGAAAC	Construction of pBACpAK-26bp	This study
**Primers to check the insertion within *cI-tetA* selection cartridge**			
ERIS	GCAAGACTGGCATGATAAGG	*cI-tetA* (reverse primer)	[1]
cI-tetA-F1	CAGCCAGCAGAGAATTAAGG	*cI-tetA* (forward primer)	[2]
**Primers for sequencing**			
IS*1*-F1	GAAATGGACGAACAGTGGGG	IS*1* extension primer	This study
IS*1*-R1	CCAGCCATCCGTCATCCATA	IS*1* extension primer	This study
IS*26*-F1	GATGGAGCTGCACATGAACC	IS*26* extension primer	This study
IS*26*-R1	GGCGCTTTATCCGTGTTGAT	IS*26* extension primer	This study

Supplementary Table 2 List of isolates and accession number in BioProject PRJNA1224291

**BioProject**	**Accession number**	**Organism**	**Isolate**
PRJNA1224291	SAMN46856268	*Escherichia coli*	ESI123-pBACpAK-16bp-3Prime-4hrTet CAT5 10
PRJNA1224291	SAMN46856269	*Escherichia coli*	ESI123-pBACpAK-16bp-3Prime-4hrTet CAT5 12
PRJNA1224291	SAMN46856270	*Escherichia coli*	ESI123-pBACpAK-16bp-3Prime-4hrTet CAT5 8
PRJNA1224291	SAMN46856271	*Escherichia coli*	ESI123-pBACpAK-16bp-3Prime-4hrTet CAT5 9
PRJNA1224291	SAMN46856272	*Escherichia coli*	ESI123-pBACpAK-16bp-3Prime-D1 ON 18
PRJNA1224291	SAMN46856273	*Escherichia coli*	ESI123-pBACpAK-16bp-3Prime-D2 5hr 4
PRJNA1224291	SAMN46856274	*Escherichia coli*	ESI123-pBACpAK-16bp-3Prime-Tet 10 4hr 5
PRJNA1224291	SAMN46856275	*Escherichia coli*	ESI123-pBACpAK-26bp-D0_ON_2
PRJNA1224291	SAMN46856276	*Escherichia coli*	ESI123-pBACpAK-26bp-D1_5hr_1
PRJNA1224291	SAMN46856277	*Escherichia coli*	ESI123-pBACpAK-26bp-D1_5hr_4
PRJNA1224291	SAMN46856278	*Escherichia coli*	ESI123-pBACpAK-26bp-D1_ON_1
PRJNA1224291	SAMN46856279	*Escherichia coli*	ESI123-pBACpAK-26bp-D2_5hr_2
PRJNA1224291	SAMN46856280	*Escherichia coli*	ESI123-pBACpAK-26bp-D2_ON_4
PRJNA1224291	SAMN46856281	*Escherichia coli*	ESI123-pBACpAK-26bp-D2_ON_5
PRJNA1224291	SAMN46856282	*Escherichia coli*	ESI123-pBACpAK-26bp-D3_5hr_1
PRJNA1224291	SAMN46856283	*Escherichia coli*	ESI123-pBACpAK-26bp-D3_5hr_3
PRJNA1224291	SAMN46856284	*Escherichia coli*	ESI123-pBACpAK-26bp-D3_5hr_6
PRJNA1224291	SAMN46856285	*Escherichia coli*	ESI123-pBACpAK-26bp-D3_ON_1
PRJNA1224291	SAMN46856286	*Escherichia coli*	ESI123-pBACpAK-26bp-D3_ON_2
PRJNA1224291	SAMN46856287	*Escherichia coli*	ESI123-pBACpAK-26bp-D3_ON_3
PRJNA1224291	SAMN46856288	*Escherichia coli*	ESI123-pBACpAK-26bp-D3_ON_4
PRJNA1224291	SAMN46856289	*Escherichia coli*	ESI123-pBACpAK-WT_D0_ON_7
PRJNA1224291	SAMN46856290	*Escherichia coli*	ESI123-pBACpAK-WT_D0_ON_8

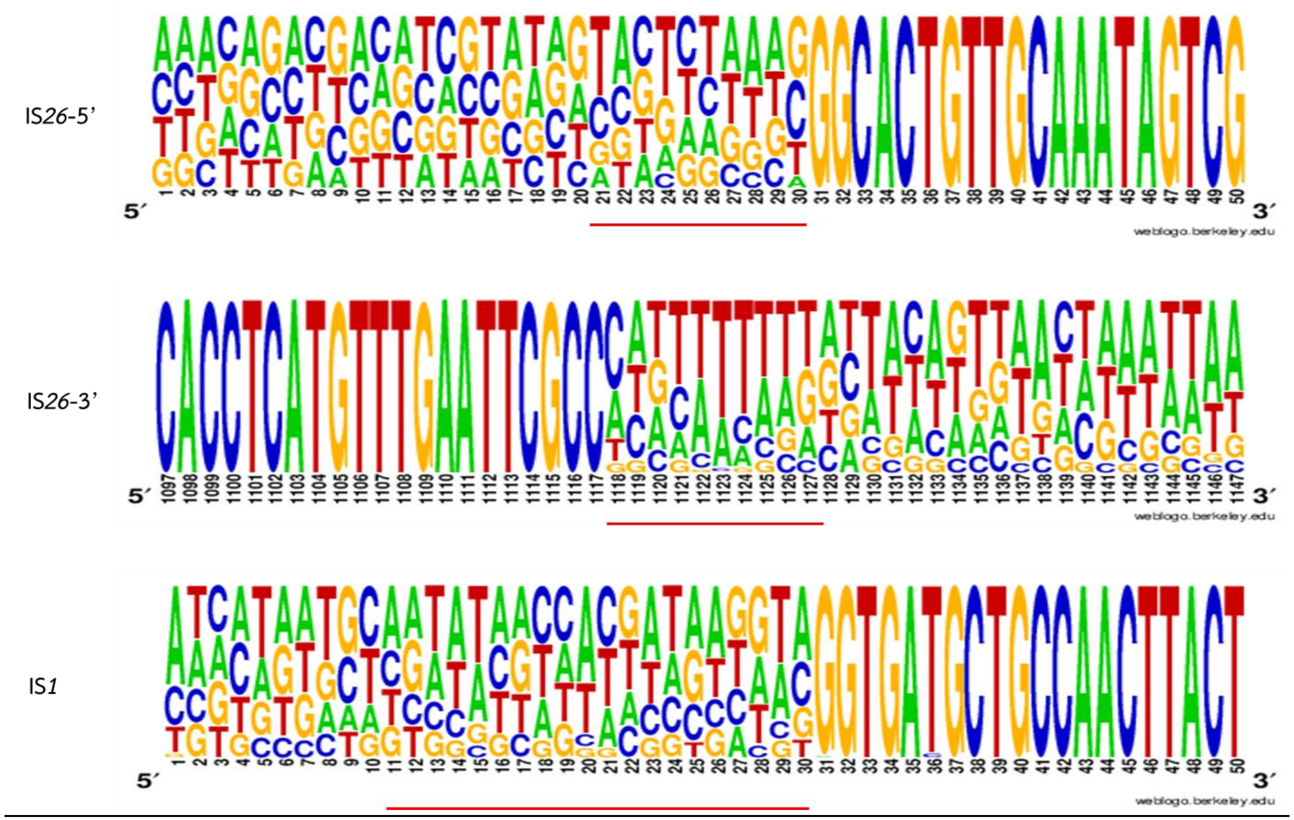

Supplementary Figure 1. Alignment of IS*26* and IS*1* insertion sites curated from the NCBI databases. The alignments were generated by aligning 120 unique insertion sites of IS*26* and 70 unique insertion sites of IS*1* with WebLogo. The red lines indicate the sequences where additional nucleotides were used in pBACpAK derivatives.

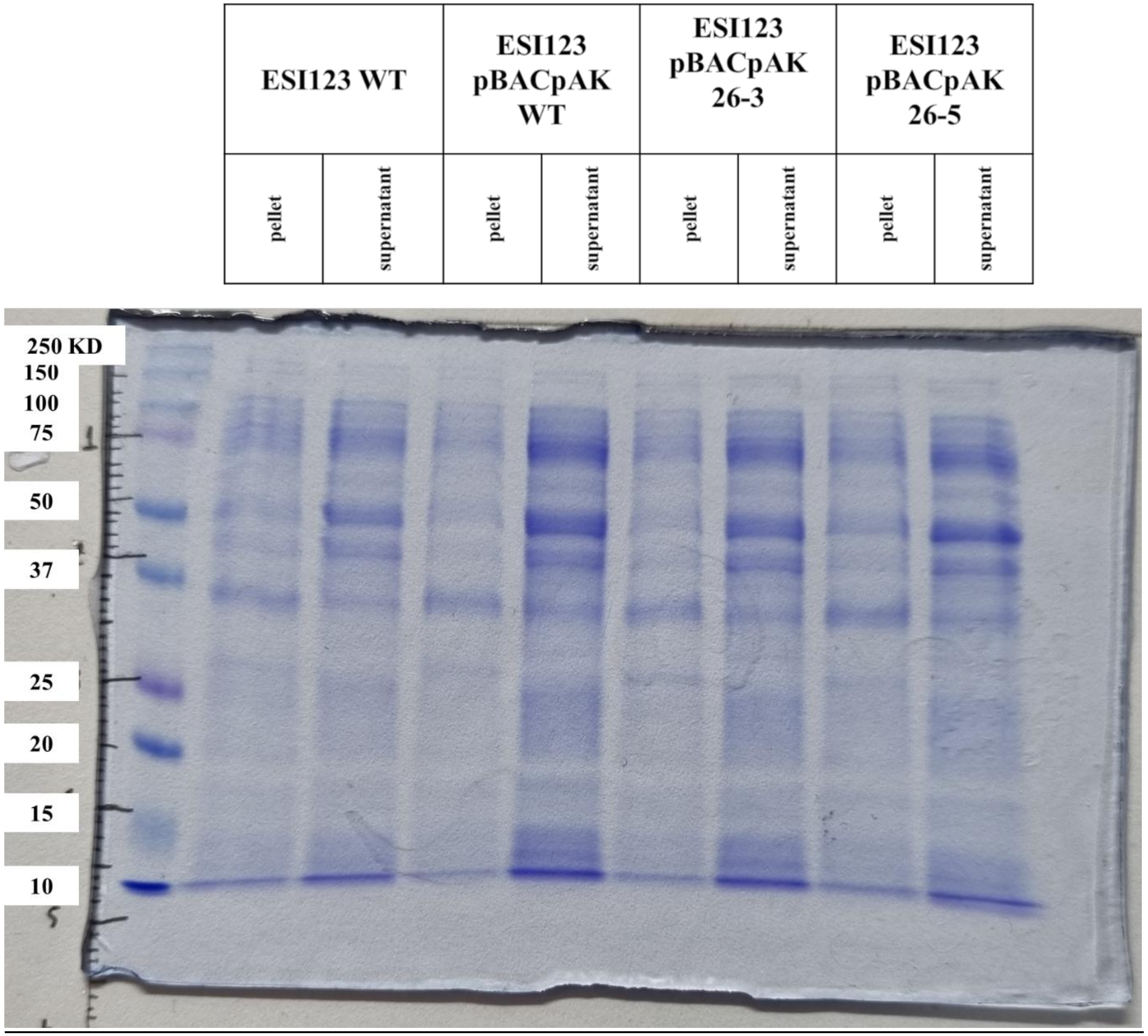

Supplementary Figure 2. SDS-PAGE analysis of whole-cell protein profiles from *E. coli* ESI123 and its derivatives. Whole-cell lysates (pellet) and culture supernatants were prepared from the wild-type ESI123, ESI123 carrying the wild-type pBACpAK vector, and ESI123 carrying the modified pBACpAK-26bp constructs (26-3 and 26-5 clones). Protein samples were separated on a 12% SDS-PAGE gel and stained with Coomassie Brilliant Blue. Molecular weight markers (left lane) are indicated in kilodaltons (kDa). No major differences in protein expression patterns were observed between strains or between pellet and supernatant fractions.

References

1. Bartosik D, Sochacka M, Baj J. Identification and characterization of transposable elements of *Paracoccus pantotrophus*. Journal of Bacteriology. 2003;185(13):3753-63. doi: 10.1128/JB.185.13.3753-3763.2003. PubMed PMID: PMC161580.
2. Tansirichaiya S, Moyo SJ, Al-Haroni M, Roberts AP. Capture of a novel, antibiotic resistance encoding, mobile genetic element from *Escherichia coli* using a new entrapment vector. J Appl Microbiol. 2021;130(3):832-42. Epub 20200917. doi: 10.1111/jam.14837. PubMed PMID: 32881179.
